# Campbell title registrations to date—February 2022

**DOI:** 10.1002/cl2.1224

**Published:** 2022-03-18

Details of new titles for systematic reviews or evidence and gap maps that have been accepted by the Editor of a Campbell Coordinating Group are published in each issue of the journal. If you would like to receive a copy of the approved title registration form, please send an email to the Managing Editor of the relevant Coordinating Group.

## CRIME & JUSTICE

Criminal justice interventions for preventing terrorism and radicalisation: An evidence and gap map

Michelle Sydes, Lorelei Hine, Angela Higginson, Laura Dugan, Lorraine Mazerolle

9 December 2021

Case management interventions seeking to counter radicalisation to violence

Sarah Marsden, James Lewis, Adrian Cherney, Martine Zeuthen, Cristina Mattei, Farangiz Atamuradova, Jürgen Brandsch, Mauro Lubrano

11 November 2021

Risk and acts of suicide and self‐harm in carceral detention of low‐ and middle‐income countries: A systematic review

Maha Aon, Marie Brasholt

17 December 2021

## CHILDREN & YOUNG PERSONS WELLBEING

Social, emotional, educational and behavioural outcomes in families and communities affected by parental incarceration: An evidence and gap map

Daragh Bradshaw, Ciara Keenan, Fiona Donson, Aisling Parkes

2 December 2021

## DISABILITY

Inclusive interventions for children with disabilities in low‐ and middle‐income countries

Anilkrishna Thota, Ebele Mogo, Dominic Igbelina, GregB Sheaf, Rahma Mustafa, Shivit Bakrania, Alberto Vasquez, Gavin Wood

27 October 2021

## EDUCATION

Key characteristics of effective preschool‐based interventions to promote self‐regulation: A systematic review and meta‐analysis

Atsushi Kanayama, Iram Siraj, Mariola Moeyaert, Natasha Robinson, Ruoying He, Rahel Warnatsch, Yuding Ding, Karou Iwasa

3 November 2021

Strategy instructions for improving short‐ and long‐term writing performance on secondary and upper secondary school students (ages 12‐19): A systematic review

André Kalmendal, Rickard Carlsson, Thomas Nordström, Ida Henriksson

15 November 2021

School‐based interventions for teachers that aim to change adolescents' aggressive behaviour: A systematic review

Marlene Paulo, Paula Vagos, Inês Leal, Ana Xavier, Nélio Brazão, Daniel Rijo

30 November 2021

The adverse effects of digital game‐based learning: A systematic review

Yu Peng, Liping Guo, Jing Liu, Kehu Yang, Lifang Li

16 December 2021

## METHODS

Assessment of outcome reporting bias in *Campbell Systematic Reviews*

Julia H. Littell, Dennis M. Gorman

1 November 2021

## SOCIAL WELFARE

Barriers and facilitators to physical activity intervention programmes among UK adults during the Covid‐19 pandemic: A systematic review

Johnson Mbabazi, Kanmodi Kehinde Kazeem, Barry Tolchard, Edward Kunonga, Lawrence Achilles Nnyanzi

25 January 2022

The barriers and facilitators experienced by homeless adults when accessing psychosocial interventions: A qualitative systematic review using thematic synthesis

Chris O'Leary, Ligia Teixeira, Esther Coren, Jordan Harrison, Harry Armitage

18 January 2022

The effectiveness of psychosocial interventions for reducing problematic substance use, improving mental health, and improving housing stability for adults experiencing homelessness: A systematic review

Chris O'Leary, Ligia Teixeira, Andrew Smith, Esther Coren, Jordan Harrison, Zsolt Kiss

18 January 2022

